# A Qualitative Examination of Stigma Among Formerly Incarcerated Adults Living With HIV

**DOI:** 10.1177/2158244016629524

**Published:** 2016-02-07

**Authors:** Holly Swan

**Affiliations:** 1 Abt Associates, Inc., Cambridge, MA, USA

**Keywords:** prison health care, HIV/AIDS, reentry, stigma, qualitative

## Abstract

The over-representation of people with stigmatized characteristics in the U.S. criminal justice population, including adults living with HIV, makes formerly incarcerated adults susceptible to multiple stigmas. Yet, the experience of HIV-related stigma, especially among individuals who have an additional compromising status in society (i.e., a criminal record) is understudied. This study used qualitative data from 30 interviews with formerly incarcerated adults living with HIV to explore the contexts within which one of these statuses becomes more salient than another for these individuals. Anticipated stigma was the primary barrier to disclosure of either status. The salience of anticipated stigma depended on the context within which disclosure may occur, including social interactions, employment, and engaging in HIV care. Communities with a high prevalence of HIV and incarceration, and health care providers within those communities should be targeted for stigma reduction efforts. Practitioners should work to empower individuals living with HIV, especially in the face of multiple potential stigmas.

## Introduction

Individuals with several characteristics that are stigmatized, or socially devalued, in contemporary U.S. society (e.g., racial/ethnic minority status, drug addiction, and psychiatric problems) are over-represented in criminal justice settings (Hartwell, 2004; Rich, Wakeman, & Dickman, 2011). Moreover, having a criminal history, or a record of incarceration, is stigmatizing in and of itself (Clear, Rose, & Ryder, 2001; Hartwell, 2004). For instance, the impact of a criminal history on employment has been long documented in the literature (Henry & Jacobs, 2007; Pager, 2003; Stoll & Bushway, 2008; Wakefield & Uggen, 2010; Western, 2002), with unemployment post-incarceration leading to re-incarceration, thus contributing to the “revolving door” of criminal justice involvement (Travis, 2005; Visher & Travis, 2003).

This intersection of stigmatizing characteristics is further complicated when someone is also diagnosed with HIV (Earnshaw, Smith, Cunningham, & Copenhaver, 2015). The prevalence of HIV in the incarcerated population of the United States is estimated to be nearly twice that of the general population (Maruschak, 2012), making individuals living with HIV who have a history of incarceration susceptible to a complex intersection of stigmatizing characteristics. This study seeks to qualitatively explore the ways that this complex intersection of characteristics is manifest in the experiences of formerly incarcerated individuals living with HIV.

Researchers and theorists interested in HIV stigma have primarily focused their energies on conceptualizing and studying the processes that lead to the creation and maintenance of stigmatizing attitudes toward people living with HIV (Earnshaw & Chaudoir, 2009; Link & Phelan, 2001; Parker & Aggleton, 2003). Although it is necessary and important to understand these processes to understand the barriers to combatting HIV, an understudied piece of the picture is whether and how people living with HIV experience this stigma, and the implications these experiences have for prevention and treatment efforts (Earnshaw & Chaudoir, 2009). These experiences among individuals who have an additional compromising status in society (e.g., a criminal record) are even less understood.

A study by Bogart and colleagues (2011) examined the effects of perceived discrimination related to a variety of stigmatizing statuses (i.e., HIV-serostatus, race/ethnicity, and sexual orientation) on mental health outcomes among Black men living with HIV (Bogart et al., 2011). These authors found that the perception of being discriminated against based on any of these statuses resulted in greater levels of depression and post-traumatic stress disorder (PTSD; Bogart et al., 2011). These mental health outcomes were worsened when the individual perceived that they were discriminated against due to all three statuses (Bogart et al., 2011). The authors conclude that greater attention needs to be paid to intersecting stigmas, particularly as they relate to prevention and treatment efforts among people living with HIV (Bogart et al., 2011).

### Conceptual Framework

Scholars have identified three distinct mechanisms through which HIV-related stigma is experienced: internalized (e.g., feeling ashamed, dirty, or unclean), anticipated (e.g., expectations of being shamed, rejected, or discriminated against), and enacted (e.g., actual rejection or discrimination; Earnshaw, Smith, Chaudoir, Amico, & Copenhaver, 2013). Research has shown that all three stigma mechanisms are an issue for HIV-positive individuals involved in the criminal justice system, and each can be a barrier to HIV care (Haley et al., 2014). In a qualitative examination of the post-incarceration linkage-to-HIV care experiences of participants in a case management randomized trial, Haley and colleagues (2014) found that participants discussed both anticipated and enacted stigma as barriers to engaging in HIV care. Specifically, participants experienced HIV-related and criminal history–related stigma in the forms of rejection by family and friends, loss of employment, and harassment. As a result of both anticipated and enacted stigma, participants did not seek HIV-related services due to stigma-related fear or shame (Haley et al., 2014). Literature has also shown that, in addition to avoidant behavior, not disclosing one's HIV status can lead to enhanced stress and mental health disorder, which may in turn worsen physical health (Bogart et al., 2011; Chaudoir, Fisher, & Simoni, 2011; Derlega, Winstead, Gamble, Kelkar, & Khuanghlawn, 2010).

Building on this literature and using the three conceptual mechanisms of HIV stigma as a framework, this analysis seeks to explore how the individuals who participated in a larger qualitative study examining the barriers and facilitators to continuous HIV care for adults with a history of incarceration manage the multiple identities that are typically stigmatized in contemporary U.S. society: Do they perceive themselves as having a stigmatized status? What are the situations or contexts within which a particular status or identity becomes more salient to them? Understanding the role of stigma in these individuals' lives can help scholars and practitioners understand the challenges these individuals face to assist them in overcoming the barriers to effective care and better health.

## Method

Data for this analysis come from 30 face-to-face semi-structured individual interviews with previously incarcerated adults living with HIV in a mid-Atlantic state. The interviews were conducted in the fall of 2011. Participants were directly recruited by staff at an urban community HIV clinic where the state's Department of Correction refers its HIV-positive clients. Inclusion criteria consisted of an HIV-positive status, previous incarceration in any of the state's correctional institutions, ages 21 and older, and English-speaking. Participants were given US$50 upon completion of the interview.

Participants were recruited through the clinical nurse coordinator (CNC) of the HIV clinic. When clinic staff identified a patient as eligible for the study, they told the CNC who then contacted the patient directly. During this initial conversation, the CNC briefly explained the nature of the project. If the patient expressed an interest in participating, an interview was scheduled. Only two patients who agreed to participate failed to show up (two additional patients were recruited in their place), and no patient who was contacted declined participation outright. All interviews followed the informed consent process as approved by the affiliated university's institutional review board.

All interviews, conducted by the lead author, were voluntary, confidential, and took place at the clinic in a private interview room to ensure as much privacy as possible (Corbin & Strauss, 2008). All participants consented to have their interviews audio recorded. Each interview participant was given a unique color coded name (e.g., Red, Spruce). The participant's name was never linked to their code as the CNC was not made aware of the codes and the researcher never documented the participants' names.

Interviews covered a variety of topics related to continuity of HIV care. The types of questions that were asked to elicit discussion around the three stigma mechanisms are listed in Table 1. The average length of the interviews was an hour and a half. After completing the interview, participants completed a brief demographic form that included their color code. All transcripts were entered into the qualitative analysis software Atlas.ti for coding and analysis.

### Analysis

Coding and analysis were approached with a mixture of deductive and inductive coding (Corbin & Strauss, 2008). Specifically, master codes (Corbin & Strauss, 2008) were created using the three stigma mechanisms of the conceptual framework as well as inductively from the interview transcripts. Secondary coding consisted of developing sub-codes (Corbin & Strauss, 2008). These sub-codes allowed for more detailed and nuanced coding within the master codes. Coding and analysis continued until saturation was reached or no new information was being discovered and codes were no longer being developed (Corbin & Strauss, 2008). Following preliminary coding, a second phase of analysis focused on refining the codebook, cleaning the coding of the transcripts, and ensuring that earlier transcripts were coded with codes developed from later transcript. Throughout analysis, memos were used to document the researcher's own reflections and to organize patterns of findings within and across interviews. Codes and memos were then utilized to uncover patterns of interconnectivity within the data to bridge the concepts into dominant themes in the final stage of analysis.

## Sample Characteristics

A breakdown of the sample characteristics is included in Table 2. The sample included 15 women, six of whom identified as White. One White woman also identified as Latina and another as Native American. Of the 22 Black participants in the sample, 13 were men, and one of these men also identified as Latino. The age range for this sample was 28 to 57 with the majority of the respondents falling in the range of 41 to 50 years. All participants resided in an urban location at the time of the interview.

All but one of the study participants were engaged in regular HIV care at the time of the interview. The participant who was not engaged in care was linked to the program's records through a drug treatment satellite of the HIV clinic. As such, she was in the pool of potential participants even though she was not receiving any regular HIV care or taking antiretroviral medications at the time of the interview. One other participant was having her CD4 and viral load counts monitored by the program, but was refusing to take medications for her HIV infection.

The majority of participants also reported receiving treatment for other health problems, indicating a high degree of co-morbidity between HIV and other health-related issues, such as diabetes and high cholesterol. The majority of the participants learned about their HIV-positive status after 1995, though some participants were diagnosed in the late 1980s/early 1990s.

Many respondents indicated that they had been in and out of prison multiple times in the past. The frequencies reported in Table 2 reflect the most recent incarceration prior to the interview. The length of the most recent incarceration ranged from less than 30 days to more than 5 years, with a majority of respondents having spent between 30 days and 6 months behind bars. A third of the respondents (*n* = 10) were released from this most recent incarceration in the same year as the interview (i.e., 2011).

## Findings

This analysis sought to answer two questions:

**Research Question 1:** Do the study participants (i.e., formerly incarcerated individuals living with HIV) perceive themselves as having a stigmatized status?
**Research Question 2:** What are the situations or contexts within which a particular status or identity becomes more salient to them?

Findings from this analysis revealed that stigma related to living with HIV and having a criminal justice background remains an issue for this group of people. For most of the participants in this study, anticipated stigma was the primary barrier to disclosure of either status. However, the salience of anticipated stigma around a particular status depended on the context within which disclosure of that status may occur: social interactions with others, employment, and engaging in HIV care. A couple of participants also discussed instances where stigma was anticipated for statuses that intersect with HIV (e.g., drug addiction) but that the participant did not identify with. These findings and their implications are discussed in detail next.

### Anticipated HIV Stigma

The anticipation of HIV-related stigma was pervasive among this study's participants. Often, the anticipation stemmed from the individual's own stigmatizing attitudes toward people living with HIV. For instance, Storm (pseudonym), a Black woman in her 40s admitted “when I didn't have [HIV] and I used to see people [who looked sick] and I'd be like `hmmm … I don't want that around me.'” Thus, participants “knew” that others must hold those same beliefs about people with HIV and so anticipated a stigmatizing response should others find out that they had HIV. One participant noted that although it was not directed at her, she had witnessed stigmatizing attitudes toward others with HIV:

I was at somebody's house, or apartment in town, and uh, somebody had come in and used the bathroom. And they didn't stay long, they left. And, after they left, somebody made the comment, hey man you better go in there and use some fuckin bleach on that toilet seat. That bitch has got the shit. And, uh, I was like, “God. That's real fucked up.” 'Cause they didn't know I was sick. (Pine, 47-year-old White woman)

The witnessing of these stigmatizing attitudes and the anticipation of being at the receiving end of the stigma prevented participants from disclosing their HIV status to others. When asked whether he told people when he was diagnosed with HIV, Olive, a Black 57-year-old man, responded,

No I didn't. I kinda kept it to myself. You know what, society is, even though you hear more and more on the radios and the TV commercials and this and that and the other, people tend to act like if I touch you, you gonna get sick or something. I mean, they're not really ready for it … it's gradually getting there, but it's not there yet. Ignorance is just, you know, it's just terrible.

This relationship between anticipated stigma and disclosure of HIV was highlighted when Olive was asked whether he had experienced people reacting in a stigmatizing way when he told them he has HIV. He replied,

Well, not to me, but I see … you never know who's listening to what, so you tend to listen to people's conversations without really getting involved in it, and you can hear the negativity … I don't even bother.

### Context: Social Interactions

In some contexts, stigma related to HIV infection was the most salient. Participants were careful to note that in certain contexts, disclosure of their HIV status was not necessary and not worth the risk of being stigmatized. For example, when discussing whether to disclose her HIV status during a drug and alcohol treatment group session, Lemon stated, “I didn't wanna share it. It's not … you know? It's not their business. I've been judged enough for it. You know? I'm not putting 'em in any harm or at any risk, so why [disclose]?” Some participants also noted that anticipated stigma in the prison setting would prevent them from disclosing to other inmates:

When I was in [jail] for my two weeks, when I was going through the process of getting my [HIV] medication and everything … they was calling me down [to the] medical [unit] so much, people was asking me like, “what's wrong wit [you]” and I just wouldn't, I didn't want to tell 'em because I felt like um, that's none of their business and I don't want them talking about me, whatever. (Neon, 31-year-old Black woman)

In most contexts, participants were mindful of keeping their health issues to themselves and therefore tended to disclose their HIV status to only a few people, particularly intimate partners, children, and/or close family members or friends. Everyone interviewed said they had disclosed their HIV status to at least one person.

Given participants' hesitance to disclose their HIV status for fear of a stigmatizing reaction, many of them were surprised that when they did disclose, rather than being stigmatized, they were received warmly, accepted, and supported. For example, when Gold, a 42-year-old White woman told her mother that she had HIV, she was met with acceptance and support,

My mom just like took it with like open arms. She wasn't like, “I don't want nuttin' to do witchu, I don't want you drinking outta my glasses, I don't want you … ” Nothing. My mom was just like, “I wanna take care of you when you get sick.”

Likewise, Sunshine told his wife and an aunt who he was close to:

[I told] my wife. Well she was my girlfriend then, but yeah I told her. She said, “well, just we gon take care of this. We gon do what we gotta do to survive.” You know. [I had] somebody in my corner. And I told my aunt. And she said “well do what you have to do to survive” and I said “yeah.” You know. And she called me, “how you doing? You make your appointments?” “Yes, ma'am.” You know. And she was there for me.

At worst, many participants found they were met with indifference. For example, when Tan, a White woman in her late forties, was asked how her family responded when she told them she had HIV, she simply stated “they didn't treat me no different.”

Some participants said that their honesty even led to heightened respect from the person they were disclosing to: “I'll let anyone know. You know. 'Cause it helps me. And it makes me stronger. You know. And then I get the upmost respect from people for being honest and being outspoken about it” (Sage, 52-year-old Black woman). As another example, Gold was nervous to tell her boyfriend because she feared he would reject her, but when she did tell him, she was surprised at his acceptance,

I should have told [my boyfriend] though, and I didn't. Because I loved him, but I didn't … I knew he would reject me, or wouldn't love me if I told him, you know? And that wasn't true, because he loved me more after I told him.

An exception to this finding was among individuals who were diagnosed with HIV in the late 80s or early 90s; these participants reported having experienced stigmatizing interactions when they were first diagnosed, but they were careful to note that things have changed. This finding is illustrated well in the following quote from Sage, a woman who was diagnosed with HIV in 1982:

Back then is when people didn't really understand what was going on [with HIV]. My family had to go through the whispering and hiding [my] plate, my forks and stuff. I've seen all that. Everybody trying to hide it … [But that has changed]. Now, I can tell … I talk to anybody about [my HIV infection]. Now I'll let anyone know [that I have HIV].

However, for a couple participants, stigmatizing reactions were still an issue in the first decade of the 21st century. Red, who was diagnosed in 2007 said “A lot of things they was doing that was hurting me, like you know I can't wash my clothes with theirs, I had to … use my own toothpaste, use my own soap.” Also, Pine, who was diagnosed in 2000 noted that “people ask me, `can I get [HIV] if I smoke after you' … [and] `my dad asked me if he could get it if he kissed me.'”

### Context: Employment

Consistent with the literature, in other contexts, such as applying for jobs, participants' criminal records topped the salience hierarchy for stigmatizing attributes. As Neon stated, “employers look at you and be like, oh she got something on her record and I really don't want her working here.” Participants' HIV status was less of a concern because potential employers “didn't know” or “didn't need to know” about their HIV. Instead, their criminal records were cited as much more salient issues than HIV. Silver, who noted that his criminal record was a concern for seeking employment stated, “HIV-positive, jobs don't look at that because you don't tell them that you're HIV-positive.” Likewise, Navy indicated, “I ain't really tell [employers] about my HIV … But my criminal record, you know, was kinda screwed. A lot of jobs do [a] background check.”

An exception to the salience of a criminal record over HIV infection with respect to employment was participants who worked in the food industry. For them, working with food heightened the salience of their HIV infection:

I just got to make sure I don't get cut, I make sure that I do what I suppose to do. That's the main thing. Not getting cut. No open wounds while I'm handling food. If I got a cut on my hand, I can't handle no food. But if I don't got no cut? I'm good.

For some participants, the uncomfortableness of working around food because of their HIV status prevented them from seeking that work. For instance, Grey, a Black man who struggles with depression noted,

Now [that I have HIV] I can't do what I … I'm not working, it's depressing. I love cooking. I had a passion for cooking. I still do. But, I don't like being around, I can't be around you know, open food like that like I really want to. You know I mean. Because you know, dealing with I might get cut, dealing with knives or anything, you know, or any type of thing. You know, I'm very careful because … I don't wish [HIV] on my worst enemy.

### Context: Engagement in HIV Care

Occasionally, the interplay of stigma related to both HIV and a criminal history created barriers to engagement in health care. Although a referral to the HIV program from Department of Correction reentry planners could potentially assist individuals returning home from incarceration in overcoming some of the challenges to reestablishing their lives in the community, knowing where to go for HIV care does not necessarily ensure that the individual will indeed connect to care. An unanticipated, though not surprising reason why people would not show up for care, even if they were referred by the prison or even had an appointment scheduled, was because of a stigma surrounding the building that the HIV clinic is housed in. For example, Magenta avoided treatment at the clinic at first and received HIV care from her private doctor. When incarceration interrupted her private insurance, however, she had to start going to the HIV clinic for treatment, but felt ashamed:

First off, I didn't want to come here. I wanted … I had my own private doctor 'cause I was on Medicaid at the time. But, eventually I had to start coming here for my treatment. And I was kind of ashamed because it's like public. More people that come here for … and, just seeing numerous people that I see every day that I would never thought had it.

Likewise, Spruce avoided HIV treatment for many years because she was embarrassed to be seen at the building where the HIV clinic is located:

And then when they see you come here, they know you got HIV, so. I used to be like, I'm trying to come in here, sneak in here … everybody in town knows … you walk in here, when you come in here, people know that you have HIV too.

One respondent, Lemon, had actually initiated care at the HIV clinic but ended up stopping her HIV care at one point because of her perception of the stigma surrounding the building, “For a little while, a couple years ago, I stopped taking meds. It was too embarrassing to come into the clinic … walking in somewhere and people knowing that you have HIV is just humiliating.” These quotes suggest that there is a perception that the actual building that the HIV clinic is housed in is the “HIV building” even though other services, such as family medicine and women's health, are housed in the same building. This perception is explicitly stated by Spruce when she says, “Everybody knows what this building is … they just know that people come here to get taken care of with their HIV.”

The HIV clinic services a large number of patients who are involved in the criminal justice system. Because of perceived stigma regarding her criminal justice status rather than her HIV status, the public nature of the HIV clinic had an interesting effect on Gold, a 42-year-old White woman who had already been a patient at the clinic when she was most recently incarcerated prior to her interview. At the time of her most recent incarceration, she was also pregnant. Because of her pregnancy, the doctor at the clinic requested that the prison allow her to be seen at the clinic so that they could continue to manage the care she had been getting prior to her incarceration:

I was in jail at the time when I was pregnant. And they ordered me to come here, [the clinic's doctor] did. Because [the doctor] knew I was HIV-positive. And they wanted me to take the medicine while I was pregnant with him. And I was shackled and handcuffed walking through that door right there. I was so mad. [laughs] And the reason why I was mad, I don't think it was so much mad, I think I was embarrassed 'cause everybody seen me.

Although it is encouraging that the community program was enforcing continued care through one of their patients' incarcerations, Gold was embarrassed because “everybody saw” that she was involved with the criminal justice system. In contrast, Magenta, Spruce, and Lemon were embarrassed about showing up at the clinic because it would announce their HIV infection. Yet, given the nature of the clinic's patient population, Spruce noted, “I've seen like three other women that we was all locked up together.” Thus, by connecting to care at the clinic, she (and the others) risked exposure of her HIV status to people she had been incarcerated with, and may potentially be incarcerated with in the future.

### Intersection of Additional Statuses

Finally, due to the overlapping nature of stigma around multiple identities that often co-occur (e.g., HIV, drug use, criminal justice involvement), the anticipation of stigma sometimes worked in the reverse direction; by nature of their HIV infection, some participants feared they would be labeled as an addict or were worried about being labeled as homosexual even though they did not identify with those statuses. For example, Sunshine, a 48-year-old Black man explained how prior to his HIV diagnosis, he stereotyped people with HIV as being homosexual, “'Cause at first I was naïve to it too, before I was diagn[osed]. [I thought] `man they must be a homosexual.' I'd stereotype too until then when [HIV] hit me and I said, `now I know I'm not [homosexual].'” Having held this stereotype previously, Sunshine was hesitant to disclose his HIV status for fear that others would stereotype him that way. Participants experienced further stress when they anticipated stigma for a status they did not consider themselves to hold:

If I had cancer, people would be so much more compassionate. 'Cause I have AIDS, um, they automatically look at you that, you know, you're a druggy. And face it. Look at me. You know? I was never a big woman to begin with. So um. That sure didn't help me. Um, I look like I'm on drugs. And I'm not! Heh. And uh, [fighting off crying] … it just makes it harder. [crying]. You know? 'Cause, people are cruel! They're really cruel. (Pine, a 47-year-old White and Native American woman)

## Discussion

This analysis attempted to untangle some of the complexities of stigma from the perspective of a group of previously incarcerated adults living with HIV. With a few exceptions, participants did not experience stigmatizing attitudes or behaviors directed at them (i.e., enacted stigma), but most participants anticipated that they would experience stigma if they disclosed that they had HIV. This anticipation was manifest through witnessing stigmatizing attitudes directed toward others and holding stigmatizing attitudes toward others with HIV prior to their own diagnosis. According to the conceptual framework used for this study, enacted stigma refers to experiencing actual rejection or discrimination (Earnshaw et al., 2013). Based on the findings from this study, however, experiencing stigma does not necessarily mean that the individual is a direct target for stigma or discrimination from someone else. Witnessing stigmatizing attitudes directed toward another who shares an identity with oneself (e.g., someone living with HIV witnessing stigma toward another person living with HIV), and holding a stigmatizing attitude toward individuals with a status (e.g., living with HIV) prior to acquiring an identity with that status (e.g., being diagnosed with HIV) may be other forms of enacted stigma. In addition, research on mental illness stigma has found that experiencing stigma around a mental illness in the past (enacted) leads one to anticipate stigma in the future (anticipated; Quinn, Williams, & Weisz, 2015). For many of the individuals in this study's sample, however, rather than being the direct recipient of rejection or discrimination, it was these nuanced forms of enacted stigma that led to anticipated stigma around that identity. Future research should explore this finding further and determine whether there are other examples or contexts where these nuances of enacted stigma and their implications are replicated.

The participants in this study need to manage stigma around multiple statuses in multiple contexts. For many participants, the best way they found to manage this complexity was to remain quiet about their health and background. However, the weight of the anticipated stigma caused distress for these individuals, often leading them to avoid situations where they may need to disclose, including seeking employment. This finding is consistent with existing literature that suggests that not disclosing one's HIV status can lead to avoidance, stress, and mental health disorder, which may in turn worsen health (Bogart et al., 2011; Chaudoir et al., 2011; Derlega et al., 2010). Moreover, the overlap of many stigmatizing attributes can create further stress, particularly if the individual does not identify with one or more of the stigmatized statuses (e.g., does not use drugs but is perceived as an addict due to his or her HIV infection). Studies have shown that clinical relationships that foster empowerment among patients improve adherence to medical treatments and can also have a therapeutic effect (Mutchler et al., 2011). Prominent HIV activist, Sean Strub (2014), has argued that stigma is a “two-sided coin”; on one side is stigma, on the other is empowerment. Focusing on empowerment in clinical relationships may help patients overcome the fear of anticipated stigma and prevent the negative effects of avoidance (Mutchler et al., 2011; Vauth, Kleim, Wirtz, & Corrigan, 2007); more research is needed on the relationship between HIV-related stigma and empowerment among this population.

Although participants tended to keep their HIV status to themselves, everyone had disclosed to one or a few people. With a couple of exceptions, most participants reported being surprised that when they did disclose, they were not met with stigma. In fact, they were supported and some were even respected for their honesty. Although most participants did not experience enacted stigma as a result of disclosing, some participants did, even as recently as 2007. Other participants explained that they knew stigma around HIV still existed through their own beliefs prior to their diagnosis, and also witnessing stigmatizing attitudes toward others with HIV. The continued prevalence of stigmatizing attitudes and the impact this has had on these individuals underscore the importance of continuing to educate people on how HIV is transmitted, the disease itself, and the risks involved. Although it seems that basic knowledge about HIV transmission and prevention is common knowledge, studies have shown that the epidemiology of low health literacy is markedly similar to the epidemiology of incarceration and HIV: minorities, individuals with less than a high school education, and individuals living in poverty tend to be more susceptible to low levels of health literacy, HIV infection, and incarceration than their demographic counterparts (Berkman et al., 2011; Delgado & Humm-Delgado, 2008; Weeks & Alcamo, 2010). In fact, a study by Adams and colleagues (2011) on the health perceptions of formerly incarcerated individuals demonstrated that the level of knowledge regarding HIV transmission and risk reduction strategies was lower than average among the participants in their study. This literature coupled with the current analysis suggests that primary prevention and education efforts in communities with a high prevalence of HIV and incarceration are necessary to reduce the amount of stigma around HIV.

As the participants articulated, it is not always necessary to disclose one's HIV status, especially when the risk of transmission is non-existent (e.g., to casual acquaintances or potential employers). In these contexts, such as applying for a job, participants' criminal record was felt to be more stigmatizing than their HIV status with the exception of employment in the food service industry where their HIV status was highly salient. One of the greatest needs of adults returning to the community from incarceration is employment (Mallik-Kane & Visher, 2008). Yet, as this analysis shows, anticipated stigma and discrimination due to a criminal history were barriers to seeking employment among this sample of participants, especially when coupled with a stigmatizing illness, such as HIV. A growing social movement called “Ban the Box” has advocated for laws prohibiting employers to ask about criminal history on job applications (Fessler, 2014; Stoll & Bushway, 2008). In this way, prospective employees are evaluated on their merits prior to discussion around criminal history (Fessler, 2014). Building on this movement, in early November 2015, President Obama directed federal agencies to “Ban the Box” on job applications (Korte, 2015). Research has indicated that these types of initiatives may not be as effective as intended, however (Stoll & Bushway, 2008); future research should continue to explore and evaluate the effectiveness of such initiatives at reducing criminal history-related stigma and consider developing additional stigma-reducing interventions that can be targeted to employers.

Finally, and consistent with the literature (Haley et al., 2014), anticipated stigma created a barrier to engaging in HIV treatment for some study participants in the context of the clinic. In most cases, the participants anticipated feeling shame and embarrassment around being seen at an HIV clinic. Another participant experienced those feelings as a result of others at the clinic learning she was involved with the criminal justice system (i.e., she arrived in handcuffs). Such feelings (shame and embarrassment) are indicators of internalized stigma (Earnshaw et al., 2013). Studies have demonstrated that internalized stigma around a mental health disorder results from anticipated stigma caused by a past experience of enacted stigma (Quinn et al., 2015). In other words, anticipated stigma mediates the relationship between enacted and internalized stigma. The current study found that the anticipation of feelings associated with internalized stigma (e.g., shame and embarrassment) can also be context dependent and related to perceived stigma around a particular place or situation. Understanding the mechanisms by which particular contexts trigger anticipation of internalized stigma (e.g., previous experiences with stigma in those situations) requires further study.

It is noteworthy that stigma in the context of engaging in HIV care was only discussed among women in this sample. Many participants anticipated stigma in other contexts, and thus often cited a concern about what others thought of them as a reason they kept their HIV status to themselves. Yet, only women reported feeling enough shame to avoid the clinic. This pattern should be explored in future research to discern whether there is a gender effect on the relationship between stigma and engagement in HIV treatment. This finding also implies that it may be beneficial for education and empowerment efforts to target women, especially in the context of engaging in HIV treatment.

### Limitations

Despite the attempt to capture diversity in experiences, there are still some sample limitations of this study with respect to the diversity of knowledge gained about the experience of stigma among HIV-positive adults. This was a small convenience sample from a single location. Moreover, all but one participant were receiving HIV care at the time of the interview, restricting the ability to draw conclusions about the role of stigma among individuals not receiving treatment. However, individuals who were already linked to care were able to provide information regarding the factors involved in some of the barriers and struggles to continuity of care that this population experiences and, importantly, techniques they used or circumstances that allowed them to overcome these problems and become engaged in care. In addition, participants in this study only resided in an urban area. Thus, differences in experiences of stigma based on geographic setting were not captured. Also, only two self-identified Latinos and one self-identified Native American participated in the study, which limited the amount of information that was learned about additional experiences of stigma among minority ethnic groups.

## Conclusion

Even if individuals with HIV do not experience enacted stigmatization from people they disclose to, the anticipation of stigma resulting from the perception of it as being “out there” and witnessing stigmatizing attitudes by others can be stressful for these individuals. The anticipation of stigma may prevent someone from disclosing their HIV status when it is important to do so. Moreover, anticipated stigma, especially around multiple and co-existing identities, can lead to avoidant behavior (e.g., avoiding social situations, not applying for jobs) that can lead to further stress and other mental health concerns that could affect treatment seeking and engagement, and ultimately result in poor health outcomes. Communities with a high prevalence of HIV and incarceration, and health care providers within those communities should be targeted for stigma reduction efforts. Public health, criminal justice, and HIV practitioners should also work to empower individuals living with HIV to take care of themselves and their communities, especially in the face of multiple potential stigmas.

## Figures and Tables

**Table 1:** Interview Questions Designed to Elicit Discussion of Stigma Mechanisms.

Interview question	Stigma mechanism
Do you tell people about your HIV status? How do the people you tell react?	Internalized, anticipated, enacted
What types of things do you do to deal with their reactions if they are negative?	Enacted
What do you think others' attitudes are (or would be) toward you if they knew your status?	Anticipated
Do you think telling someone your status would affect how they treated you? In what ways?	Internalized, anticipated
How have your relationships with others affected how comfortable you are telling others that you are HIV-positive?	Internalized, enacted
How have others' attitudes toward you influenced your HIV treatment?	Internalized; enacted
Do other parts of your circumstances, such as having a criminal record, substance use history, influence things like employment, housing, financial assistance? in what ways?	Intersecting, internalized, anticipated, enacted
Do other parts of your circumstances, such as having a criminal record, substance use history, influence the way others treat you? In what ways?	Intersecting, internalized, anticipated, enacted

**Table 2:** Sample Characteristics (*N* = 30).

Demographic	*n*
Gender	
Male	15
Female	15
Race	
Black	22
White	8
Ethnicity	
Latino	2
Native American	1
Age	
25–40	6
41–50	20
51–60	4
Residence	
Urban	30
Receiving treatment for	
HIV/AIDS	29
(Not on antiretroviral medication)	2
Mental/emotional issues	12
Alcohol/drugs^a^	4
Other physical problems	8
Two of the above	12
All of the above	5
Year tested HIV+	
2005–2010	7
2000–2004	9
1995–1999	8
1980–1994	6
Testing site	
Prison/jail	10
Community center	13
Hospital or doctor's office	7
Length of incarceration	
Less than 30 days	2
30 days-6 months	11
7 months-1 year	6
1.5–5 years	6
More than 5 years	5
Year of release^b^	
2011	10
2005–2010	14
2000–2004	1
1990s	2

a Although only four respondents indicated that they were receiving treatment for alcohol or drug problems at the time of the interview, 83% (*n* = 25) of participants discussed a history of alcohol or drug dependence during their interviews.

b Three respondents did not disclose or could not remember their release date, so only 27 respondents are counted.
